# Reply

**DOI:** 10.1016/j.xops.2025.100841

**Published:** 2025-06-03

**Authors:** Hemal P. Patel, Sandra Stinnett, Peter Weng, Cason B. Robbins, Jamie J. Karl, Lejla Vajzovic, Sharon Fekrat

**Affiliations:** Department of Ophthalmology, Duke University School of Medicine, Durham, North Carolina

Thank you for your comments in response to our recent article. We appreciate your interest in the methodology and herein provide a thoughtful response to each point raised.

First, we agree that overemphasizing the difference between final and presenting visual acuity (VA) can be misleading.^1^ Vickers and Altman focus on 2 approaches: using either the final value (i.e., VA) or the change in value as the outcome. They recommend adjusting for the initial value when the final value is the outcome but do not address whether or how to include the initial value when modeling the change in values. In our analysis, we modeled for change in VA (final minus presenting VA) while adjusting for presenting VA. This is equivalent to modeling final VA with presenting VA as a covariate (only the coefficient on presenting VA decreases by 1), while other effect estimates, including on antithrombotic status, remain unchanged. This equivalence is demonstrated mathematically below, based on the structure used in Vickers and Altman. Statisticians generally agree that both approaches are valid, especially when presenting VA is not the primary variable of interest but rather serves as a control.^2^

**Proof of equivalence:**

Given a simplified model (per Vickers and Altman):

y−x=ax+bz+c

where:

• *y* = final VA,
• *x* = presenting VA,
• *y–x* = change in VA,
• *z* = antithrombotic status,
• *a* = effect of *x* on *y*,
• *b* = effect of *z* on *y*,
• *c* = constant.

Add *x* to both sides:

y=ax+x+bz+c

Group and factor *x*:

y=(a+1)x+bz+c

Thus, switching the outcome from change in VA (*y–x*) to final VA (*y*) shifts only the coefficient on presenting VA by +1. Other predictors, including antithrombotic status, remain unchanged.

Second, table 2 shows treatment differences by antithrombotic group with no more than 10–20% point variations in use of pneumatic displacement, vitrectomy, or intravitreal anti-VEGF injection between each antithrombotic group. Chi-square analysis was not possible due to overlapping treatments (as is common in clinical scenarios). Instead, each treatment was included as an independent indicator variable, allowing the models leeway in assigning significance without imposing linear relationships. Interaction terms between these independent predictors were considered but ultimately avoided due to the risk of bias from small sample sizes. ^3^ In 4 regression models, treatment choices were generally not a significant predictor of change in VA, except for pneumatic displacement in the antiplatelet group, controlling for submacular hemorrhage (SMH) size. Treatment choices were not our primary focus and thus were not discussed further.

Third, we appreciate the interest in the duration of antithrombotic use, but electronic medical records were inconsistently updated at each clinic visit, limiting longitudinal analysis. That said, we believe that not incorporating a longitudinal assessment had minimal impact on validity. Most VA loss from SMH occurs early in its course as blood clots, fibrin contracts, and photoreceptor shearing occurs.^4^ Recombinant tissue plasminogen activator is often given promptly (and only once) for clot lysis and prevention of further blood clotting.^5^ Therefore, the mechanistic similarities of anticoagulants and antiplatelet agents to recombinant tissue plasminogen activator will mostly, if not entirely, be realized when the hemorrhage initially occurs. Thus, antithrombotic use at presentation with SMH is the most relevant variable. That said, although most patients cannot safely discontinue systemic antithrombotic medications without morbidity and mortality risk, future prospective studies could explore the impact of continued use versus short-term cessation on VA outcomes.

We thank Drs Pierre-Henry and Creuzot-Garcher for their interest in our paper. We believe our analytical methods and interpretation are sound and do not reflect residual confounding or overinterpretation of VA gains. Instead of recommending alternate treatment plans, we discussed our results at a single institution regarding the impact of various antithrombotic medications in eyes with SMH due to wet age-related macular degeneration and suggested further investigation. We appreciate the opportunity to engage in discourse with the broader scientific community regarding our work.
